# Modeling for High-Frequency Spurious Responses in Incredible High-Performance Surface Acoustic Wave Devices

**DOI:** 10.3390/mi15010134

**Published:** 2024-01-15

**Authors:** Guanzhen Jiang, Yao Shuai, Zijie Wei, Jialin Yao, Wenbo Luo, Xinqiang Pan, Chuangui Wu, Wanli Zhang

**Affiliations:** 1 School of Integrated Circuit Science and Engineering, University of Electronic Science and Technology of China, Chengdu 611731, China; jgz312182209@163.com (G.J.); rivalry970929@163.com (Z.W.); y1067960670@126.com (J.Y.); luowb@uestc.edu.cn (W.L.); panxinqiang@uestc.edu.cn (X.P.); cgwu@uestc.edu.cn (C.W.); wlzhang@uestc.edu.cn (W.Z.); 2 State Key Laboratory of Electronic Thin Films and Integrated Device, University of Electronic Science and Technology of China, Chengdu 611731, China

**Keywords:** I.H.P. SAW, Carrier Aggregation, high-frequency spurious responses, extended COM model

## Abstract

To ensure that surface acoustic wave (SAW) filters fulfill the requirements of Carrier Aggregation (CA) applications, the development of modeling tools that can forecast and simulate high-frequency spurious responses has been necessary. This paper presents an advanced methodology for extending the coupling-of-modes (COM) model to obtain precise modeling of the high-frequency spurious responses of incredible high-performance surface acoustic wave (I.H.P. SAW) devices. The extended COM (ECOM) model is derived by modifying the conventional COM model and extending it accordingly. The parameters used in this model are determined through numerical fitting. For validation, firstly, the ECOM model is applied to a one-port synchronous I.H.P. SAW resonator, and the simulation and measurement results match. Then, the structural parameters of the ECOM model are varied, and the accuracy of the model after the structural parameters are varied is verified. It is demonstrated that this model can be applied to the design work of SAW filters. Finally, the ECOM model is applied to the design of the I.H.P. SAW filter based on a 42°YX-LiTaO_3_ (LT)/SiO_2_/AlN/Si structure. By using this method, the I.H.P. SAW filter’s high-frequency spurious response can be predicted more accurately.

## 1. Introduction

In recent decades, there has been a rapid emergence of wireless communication systems, including mobile communications (CDMA, UMTS, GSM, etc.), global positioning systems (GPSs), data transmission systems (such as WLAN, Bluetooth, etc.), satellite communication and other military communication systems [1,2]. Notably, significant advancements have been achieved in mobile communications, wherein radio frequency (RF) devices, particularly surface acoustic wave (SAW) devices, serve as fundamental components. The evolution of mobile phone systems has catalyzed increasing interest in SAW devices. And with the development of the fifth-generation (5G) mobile communication technology, the technical requirements for SAW devices are also constantly improving [3,4]. SAW filters are widely used in home TV, mobile communications, radio frequency filters and radar due to their simple structure, few mask layers, easy miniaturization and low cost [5]. Through the continuous improvement in communication technology, SAW devices have been developed into various high-performance acoustic filters with the support of some new architectures, new materials and advanced modeling techniques.

Recently, SAW technologies have been significantly enhanced by the introduction of wafer-bonding technology such as the incredible high-performance (I.H.P.) surface structure. The multilayered structure of I.H.P. SAW devices confines SAW energy in the surface area of the substrate, resulting in an extremely high quality (Q) factor, a low temperature coefficient of frequency (TCF), and an improved electromechanical coupling coefficient (K^2^) [6,7,8,9,10,11,12]. Nonetheless, with increasingly stringent performance criteria, high-frequency spurious modes have emerged as critical challenges for such devices, deteriorating the high-frequency out-of-band rejection capacity of I.H.P. SAW devices.

Substantial research has been conducted to understand the spurious responses adjacent to the passband. In particular, scalar potential theory was used to study transverse mode characteristics, and various design methodologies were developed based on this theory to eliminate transverse spurious modes [13]. For instance, M. Solal proposed that by changing the layout of the transducer, it was possible to improve guidance and obtain a “piston mode” that suppressed transverse modes. Hideki Iwamoto concluded that a tilted resonator with a small tilt angle was free of spurious responses while maintaining a high Q factor [14]. Due to the dispersive nature of I.H.P. SAW devices, the piston mode does not provide broadband energy confinement and scattering loss suppression. Therefore, Wong et al. designed a new transverse edge with a double busbar for I.H.P. SAW devices. This structure provides not only broadband energy limitation but also transverse mode suppression [15]. In addition, there is a need to be able to achieve fast and accurate simulation of the spurious response to provide a basis for the elimination of the spurious response. This places higher demands on the design model, requiring modification and extension of the design model. The coupling of two modes in SAW devices results in the generation of new spikes, and the traditional single-mode coupling-of-modes (COM) model cannot consider the coupling of two modes. Therefore, some researchers have proposed the multi-mode COM model and the multi-mode P-matrix model [16,17,18,19], which can take the coupling between two modes into account and thus enable the fitting analysis of spurious modes.

However, in response to the increase in mobile data traffic, frequency bands for Long-Term Evolution (LTE), standardized by the Third Generation Partnership (3GPP), have dramatically increased. Furthermore, LTE and LTE-Advanced now have tightly allocated bands in their spectra [20,21]. Several systems have also been introduced, such as Carrier Aggregation (CA) for uplink and High-Power User Equipment (HPUE) for service delivery [22]. Consequently, in designing filters to integrate new functionalities and frequency bands, it becomes imperative to address not only the spurious responses near the passband but also those manifesting at higher frequency ranges. Simultaneously, the prevalence of multiple high-frequency resonances within the I.H.P. SAW resonator presents considerable challenges to multi-mode COM modeling.

In this study, we introduce a novel model, fundamentally based on the COM framework. To achieve this, we have extended the COM model with a modified phenomenological model. This model enables the high-frequency spurious response of I.H.P. SAW devices to be analyzed more easily and efficiently. First, we analyzed the effect of the high-frequency spurious resonance of the SAW resonator on the ladder-type SAW filter using the conventional single-mode COM model. Subsequently, we have proposed the extended COM (ECOM) model, based on the conventional single-mode COM model, for analyzing the high-frequency spurious responses of I.H.P. SAW resonators. In order to verify the validity of this method, we fabricated one-port synchronous SAW resonators with different structural parameters and compared them with the simulation results of the ECOM model. Finally, we applied the proposed model to the design of a ladder-type SAW filter and conducted a comparative analysis with the measured results, which proved to be quite gratifying.

## 2. Experimental Method

### 2.1. Characteristics of I.H.P. SAW Devices

**Figure 1:**
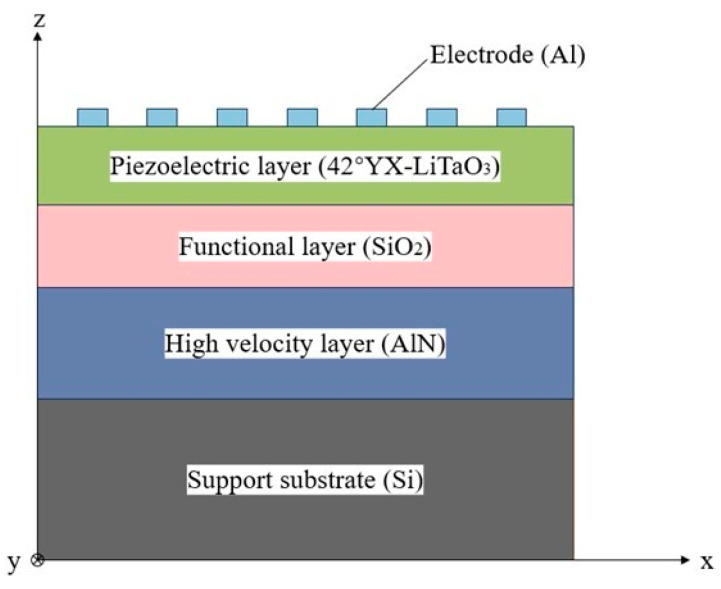
Basic structure of incredible high-performance surface acoustic wave (I.H.P. SAW) device.

The basic structure of the I.H.P. SAW device (University of Electronic Science and Technology of China, Chengdu, China) has a multi-layer construction with thin materials on a substrate. A thin piezoelectric layer, a functional layer and a higher-velocity layer are attached to a support substrate [9]. Figure 1 depicts the fundamental architecture of I.H.P. SAW devices, wherein 42°YX-LiTaO_3_ (LT) serves as the thin piezoelectric layer, SiO_2_ as the functional layer, AlN as the higher-velocity layer, and aluminum as the electrode material.

**Figure 2:**
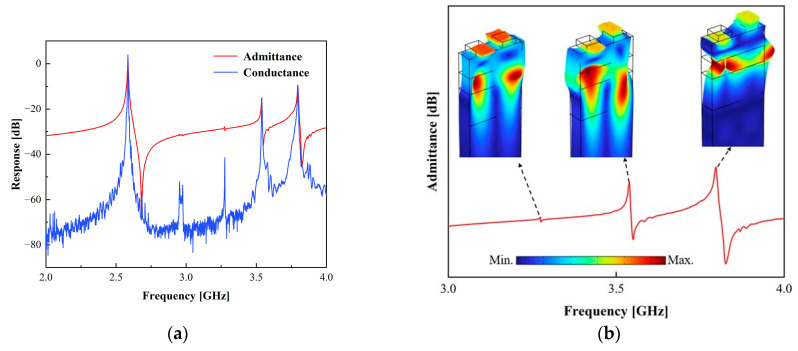
(**a**) Measured admittance and conductance characteristics of one-port synchronous SAW resonators. (**b**) Displacement schematic for high-frequency spurious responses.

Based on Figure 1, observe the stack configuration and parameters: $h_{Al}=0.08\lambda$, $h_{LT}=0.3\lambda$, $h_{SiO_{2}}=0.3\lambda$, $h_{AlN}=0.4\lambda$, where λ stands for the SAW wavelength propagating on the surface [9]. Based on the above structure and parameters, we measured the admittance and conductance response of the one-port synchronous SAW resonator. Figure 2a shows the measured resonator admittance and conductance responses. The figure shows a significant number of spurious responses at the high frequency of the I.H.P. SAW resonator. To delve deeper into the high-frequency spurious responses, Figure 2b exhibits the displacement schematics of each high-frequency spurious resonance. The displacement schematics reveal that the acoustic modes at 3.3 GHz and 3.539 GHz exhibit the traits of high-order shear-horizontal (SH) mode, with the mode at 3.539 GHz demonstrating a more pronounced response. Furthermore, the acoustic mode at 3.807 GHz is characterized by the Sezawa mode, manifesting the most substantial response [23].

### 2.2. Traditional Single-Mode COM Model

**Figure 3:**
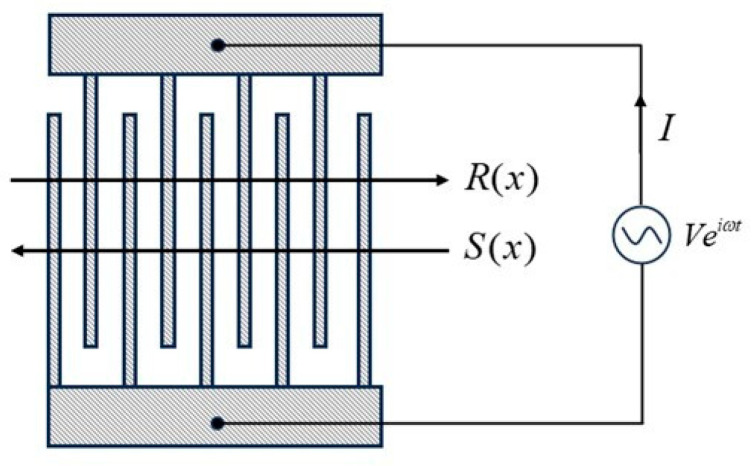
Illustration of the coupling-of-modes (COM) model.

In SAW device design, the COM model has been successfully used for the modeling of SAW devices for many years. In this paper, we adopted the COM formulation presented by Plessky [24]. Figure 3 illustrates the basic structure of the SAW transducer, the response of which can be characterized in terms of current as well as emitted wave amplitude. It is assumed that there are two acoustic wave modes propagating in opposing directions within an infinite-length periodic grating array. The rightward propagating acoustic surface wave, R(x), generates an echo, S(x), which then propagates in the opposite direction due to the modulation of the periodic grating structure. The two modes couple and affect each other during the propagation process. Furthermore, acoustic waves within the transducer are stimulated by applying voltage *V* through an excitation source connected to the bus of the transducer. The surface electrodes’ presence modifies the acoustic surface wave velocity at the free surface and introduces a coupling between the modes [24]. Acoustic damping results in a slight acoustic loss. A linear coupling between the amplitudes, voltage, and current exists, and therefore, the coupling of modes equation takes the given form:

(1) $$\{\begin{array}{c} \frac{dR(x)}{dx}=-i\delta R(x)+i\kappa S(x)+i\alpha V\\ \frac{dS(x)}{dx}=-i\kappa^{*}R(x)+i\delta S(x)-i\alpha^{*}V\\ \frac{dI(x)}{dx}=-2i\alpha^{*}R(x)-2i\alpha S(x)+i\omega CV \end{array}.$$

where R(x) and S(x) correspond to the slowly varying fields of forward- and backward-propagating SAWs, respectively, κ is the reflection coefficient, C is the capacitance parameter, α stands for transduction coefficient, δ is the detuning parameter, which is defined by the following:

(2) $$\delta =\frac{\omega }{v}-\frac{2\pi }{p}-i\gamma .$$

where v is SAW velocity, *p* is the period of the fingers, γ is the attenuation parameter. In the conventional single-mode COM equation, there are five parameters to be determined, which include κ, α, γ, v, C. As these parameters relate to materials, they are known as material parameters.

**Figure 4:**
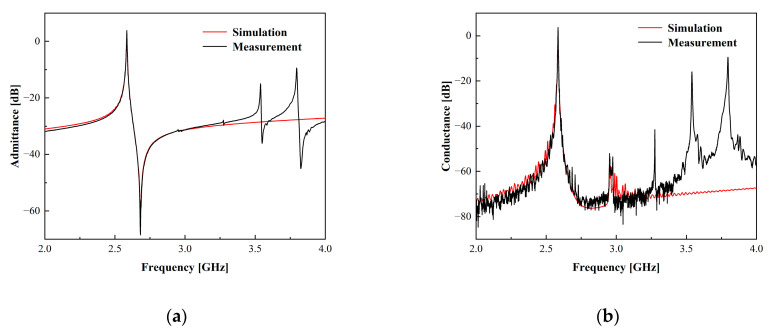
Comparative results of simulations using the COM model and measurements of one-port synchronous SAW resonator. (**a**) Simulated and measured admittance characteristics of a resonator. (**b**) Simulated and measured conductance characteristics of a resonator.

By utilizing the traditional single-mode COM model outlined in this section, a simulation of a one-port synchronous resonator can be achieved, leading to the calculation of the resonator’s admittance in relation to frequency. Shown in Figure 4 is the result of simulated and measured admittance and conductance of a one-port synchronous SAW resonator. The structural parameters for both the simulated and measured one-port synchronous SAW resonator are provided in Table 1. These structural parameters consist of the period of the fingers *p*, wherein the SAW wavelength corresponds to the IDT’s period *p*, aperture W, the number of IDT finger pairs $N_{t}$, as well as the number of reflector gratings $N_{g}$, metallic ratios α.

The comparison depicted in Figure 4 demonstrates a fundamental alignment between the admittance and conductance curves, inclusive of the principal resonance domain of the one-port synchronous SAW resonator and the spurious responses proximal to the anti-resonance frequency, but it cannot be fitted above 3 GHz due to the fact that the accuracy of the conventional single-mode COM model is essentially limited to a narrow band of frequencies around the principal resonance. Since body waves appearing at high frequencies produce spurious responses, this leads to an inability to accurately predict high-frequency out-of-band rejection at the filter design stage, which results in a large error between the simulation and measurement of the filter. Therefore, the requirements of SAW filter performance are not limited to the passband of the device, but typically cover a wider range of frequencies, so a few simple extensions can be introduced into the COM model to further improve agreement with the measurements.

### 2.3. ECOM Model

As highlighted in the preceding section, the traditional single-mode COM model exhibits specific constraints in the design of I.H.P. SAW devices. Not only must we simulate the resonator with accuracy, but we must also guarantee that the results of simulation and measurement remain consistent while adjusting the structural parameters of the resonator. Therefore, we would like to use the phenomenological model, which is similar to the COM model, to fit the spurious response at high frequencies, and take advantage of phenomenological modeling to reduce errors when changing structural parameters. A phenomenological model is a physical model that explains a physical phenomenon without considering its intrinsic causes. The model is obtained by generalizing experimental facts. A multitude of phenomenological models have been proposed for the modeling and analysis of high-performance SAW devices. These include the COM model, the P-matrix model, equivalent circuit models, and the angular spectrum of waves model [24,25]. Since the COM theory is phenomenological, the values of COM parameters cannot be determined from the theory itself, but must be introduced outside. If the phenomenological model is used correctly and with accurate parameters, it provides relatively accurate simulation results [24]. It is apparent that the phenomenological model exhibits considerable sensitivity to parameter selection. When changing the parameters, the simulation results of the phenomenological model can be as close as possible to the actual measurement results. In this paper, the advantages of the phenomenological model are used to extend the traditional single-mode COM model so as to ensure the accuracy of the model when changing the structural parameters. Consequently, by modifying and updating the single-mode COM model and extending the COM model to some extent, we have successfully derived the ECOM model.

**Figure 5:**
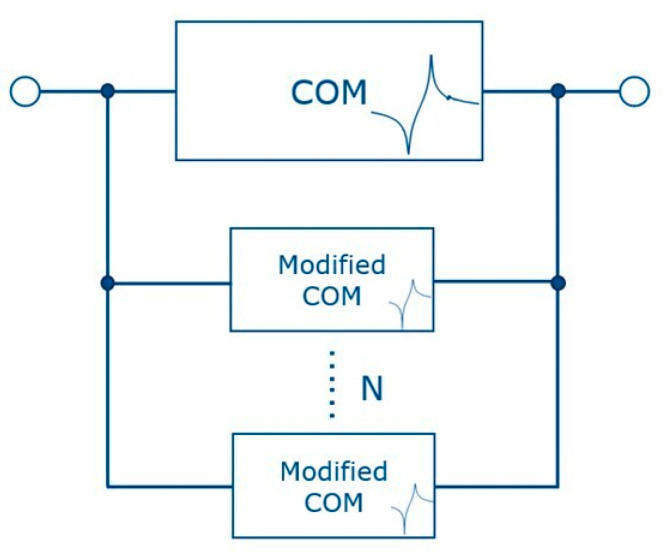
The extended COM (ECOM) model.

Figure 5 shows the schematic of the ECOM model in the SAW devices design implementation, where the N is assumed to be the number of the high-frequency spurious responses to be analyzed.

The ECOM model comprises two distinct components: the conventional single-mode COM model simulating the main resonance, and the modified COM model tailored for high-frequency resonance simulations. Both models are phenomenological models, and when cascaded, the resultant ECOM model retains its phenomenological essence. Consequently, the ECOM model preserves the advantageous sensitivity to parameter variations inherent to phenomenological models.

**Figure 6:**
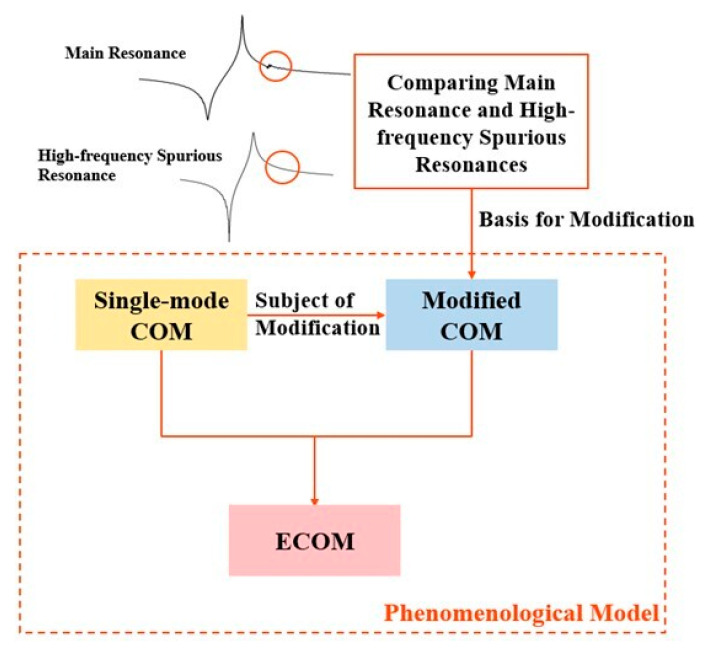
The process of realization of the ECOM model.

To realize the simulation of the ECOM model, it is necessary to compare the main resonance with the high-frequency spurious resonance. The difference between the two is the presence of spurious waves in the main resonance region that are slightly higher than the anti-resonance frequency. To simulate the high-frequency resonance, modification of the traditional COM model is imperative. After obtaining the modified COM model, the traditional single-mode COM model and the modified COM model are cascaded to obtain the ECOM model. The specific implementation flowchart of the ECOM model is shown in Figure 6, which demonstrates the relationship between the single-mode COM model, the modified COM model, the ECOM model, and the phenomenological model, to avoid conceptual confusion.

For one-port synchronous SAW resonators, the modeling of the COM model and the modified COM model must introduce the P-matrix. The P-matrix is a matrix of sound wave–signal, excitation–response relationships at the left and right positions of the interdigital transducers (IDTs) obtained by solving the COM equations. The main reason for introducing the P-matrix is that it is convenient to use it for modeling devices consisting of several different substructures, such as the reflector gratings, IDTs, and gaps that make up a one-port synchronous resonator. The P-matrix takes the specific form [24]:

(3) $$\left[\begin{array}{c} \varphi_{-}(x_{1})\\ \varphi_{+}(x_{2})\\ I \end{array}\right]=\left[\begin{array}{ccc} P_{11} & P_{12} & P_{13}\\ P_{21} & P_{22} & P_{23}\\ P_{31} & P_{32} & P_{33} \end{array}\right]\left[\begin{array}{c} \varphi_{+}(x_{1})\\ \varphi_{-}(x_{2})\\ V \end{array}\right].$$

This matrix considers a transducer located in the region $x=\left[x_{1},x_{2}\right]$. The response of the structure may be characterized by the current *I* and the amplitudes $\varphi_{+}(x_{2})$ and $\varphi_{-}(x_{1})$ representing the waves launched by the structure. The 2 × 2 submatrix in the upper left corner describes the scattering of waves that enter the structure from the outside. It includes the reflection coefficients ($P_{11}$ and $P_{22}$) and the transmission coefficient ($P_{12}=P_{21}$). The remaining parts of the P-matrix pertain solely to the transducers. The components $P_{13}$ and $P_{23}$ describe the excitation efficiency of the IDT, while the components $P_{31}$ and $P_{32}$ measure the current generated in the IDT by the incoming waves [24].

By solving the COM equation, the specific expression for the P-matrix is obtained as in [24]:

(4) $$\begin{array}{l} P_{11}=\frac{i\kappa^{*}\sin(qL)}{q\cos(qL)+i\delta \sin(qL)}\\ P_{12}=\frac{{\left(-1\right)}^{N_{t}}q}{q\cos(qL)+i\delta \sin(qL)}=P_{21}\\ P_{22}=\frac{i\kappa \sin(qL)}{q\cos(qL)+i\delta \sin(qL)}\\ P_{13}=-L\frac{\sin(qL/2)}{qL/2}\frac{\left(\delta \alpha^{*}+\kappa^{*}\alpha )\sin(qL/2)-i\alpha^{*}q\cos(qL/2\right)}{q\cos(qL)+i\delta \sin(qL)}\\ P_{23}=-{\left(-1\right)}^{N_{t}}L\frac{\sin(qL/2)}{qL/2}\frac{\left(\delta \alpha +\kappa \alpha^{*})\sin(qL/2)-i\alpha q\cos(qL/2\right)}{q\cos(qL)+i\delta \sin(qL)}\\ P_{31}=-2P_{13}\\ P_{32}=-2P_{23}\\ P_{33}=i\omega CL-L4i\frac{\delta {\left|\alpha \right|}^{2}+ℜ(\kappa^{*}\alpha^{2})}{\delta^{2}-{\left|\kappa \right|}^{2}}-\frac{4}{q^{3}}\frac{\left((\delta^{2}+{\left|\kappa \right|}^{2}){\left|\alpha \right|}^{2}+2\delta ℜ(\kappa^{*}\alpha^{2}))(1-\cos(qL)\right)}{q\cos(qL)+i\delta \sin(qL)}+\frac{4i}{q^{3}}\frac{\left(\delta {\left|\alpha \right|}^{2}+ℜ(\kappa^{*}\alpha^{2}))q\sin(qL\right)}{q\cos(qL)+i\delta \sin(qL)}. \end{array}$$

where $q=\sqrt{\delta^{2}-{\left|\kappa \right|}^{2}}$ is slowly varying wavenumber, $L=N_{t}\times p$ is the length of the IDT, *p* is the period of the fingers, $N_{t}$ is the number of IDT finger pairs, ω is given by ω=2πf, in which f is the frequency of the wave.

Since the one-port synchronous resonator is composed of substructures, the whole device can be modelled by connecting the acoustic and electric inputs and outputs by treating each substructure independently and describing it using a P-matrix based on the assumption of locality [26,27]. Consider two substructures, *A* and *B*, which share a common acoustic port and are electrically connected in parallel. Their structure can be described using two P-matrices, $P^{A}$ and $P^{B}$, respectively. The cascade rule for P-matrices is expressed as follows [28,29]:

(5) $$\begin{array}{l} P_{11}=P_{11}^{A}+P_{11}^{B}\frac{P_{21}^{A}P_{12}^{A}}{1-P_{11}^{B}P_{22}^{A}}\\ P_{12}=\frac{P_{12}^{A}P_{12}^{B}}{1-P_{11}^{B}P_{22}^{A}}=P_{21}\\ P_{22}=P_{22}^{B}+P_{22}^{A}\frac{P_{12}^{B}P_{21}^{B}}{1-P_{11}^{B}P_{22}^{A}}\\ P_{13}=P_{13}^{A}+P_{12}^{A}\frac{P_{13}^{B}+P_{11}^{B}P_{23}^{A}}{1-P_{11}^{B}P_{22}^{A}}\\ P_{23}=P_{23}^{B}+P_{21}^{B}\frac{P_{23}^{A}+P_{22}^{A}P_{13}^{B}}{1-P_{11}^{B}P_{22}^{A}}\\ P_{33}=P_{33}^{A}+P_{33}^{B}+P_{32}^{A}\frac{P_{13}^{B}+P_{11}^{B}P_{23}^{A}}{1-P_{11}^{B}P_{22}^{A}}+P_{31}^{B}\frac{P_{23}^{A}+P_{22}^{A}P_{13}^{B}}{1-P_{22}^{A}P_{11}^{B}}. \end{array}$$

**Figure 7:**
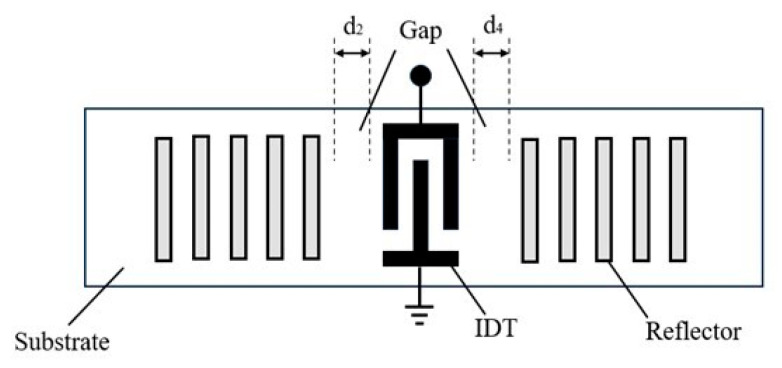
Schematics of the one-port SAW resonator.

The admittance of the one-port synchronous SAW resonator is represented using the components of the P-matrix. A schematic diagram of a one-port synchronous SAW resonator is shown in Figure 7. It consists of an IDT and two reflector gratings on either side. The distance between the IDT and the left and right reflector gratings are denoted by $d_{2}$ and $d_{4}$, respectively. The IDT converts an externally supplied electrical signal into an acoustic surface wave that travels in the ±x directions. The wave is then reflected back to the IDT by the metal reflector gratings, forming a resonant cavity. To enhance the reflection, the metal reflector gratings are typically short circuited [26]. The reflection coefficient Γ of the reflector gratings can be obtained from the P-matrix of the IDT by setting V=0. It is evident that the reflection coefficient is $P_{11}$ when the sound wave is incident from the left side of the reflector gratings, and $P_{22}$ when it is incident from the right side.

**Figure 8:**
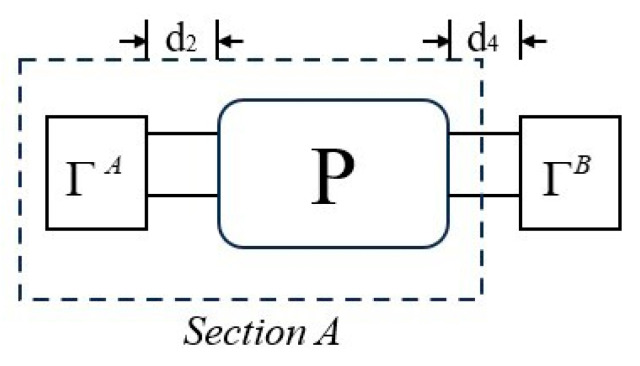
Simplified block diagram of the one-port SAW resonator.

Figure 8 displays a simplified form of the one-port resonator structure, where $\Gamma^{A}=P_{22}e^{-2i\kappa d_{2}}$ and $\Gamma^{B}=P_{11}e^{-2i\kappa d_{4}}$. Derive the admittance *Y* of the one-port synchronous resonator using the reflection coefficient formula and the cascade formula for the P-matrix:

(6) $$Y=P_{33}^{A}+\frac{\Gamma^{B}P_{32}^{A}P_{23}^{A}}{1-\Gamma^{B}P_{22}^{A}},$$

where the P-matrix of section *A* is obtained by cascading the P-matrix of the left reflecting gratings and the P-matrix of the IDT. The simulation results of the single-mode COM model can be obtained by using Equation (6) and the COM parameters.

After simulating the main resonance with the conventional single-mode COM model, it is necessary to modify the model to enable simulation of high-frequency spurious resonance. Upon comparing the main resonance and high-frequency resonance waveforms in Figure 6, it is evident that the main resonance contains spurious waves at frequencies higher than the anti-resonance frequency, which are absent in the high-frequency resonance waveform. The modified COM model can be derived by removing the spurious response slightly above the anti-resonance frequency from the COM model. Beginning with the fundamental principle of one-port resonator operation, the IDT excites an acoustic wave and enters a resonant state when the frequency of the input RF signal is equal to the ratio of the resonator’s equivalent surface acoustic velocity to the period of the finger. At this point, a standing wave is generated between the fingers. Part of the wave is reflected between the fingers, and the rest is reflected back to the IDT region by short-circuiting reflector gratings. The majority of the energy is confined within the IDT and reflector gratings [30]. This spurious response is generated by the anomalous dispersion due to BAW backscattering [31,32]. We can cut the total energy confined in the IDT by reducing the standing waves due to reflection from the reflector gratings. The reduction in the total energy will reduce the degree of backscattering, thus achieving the removal of spurious response. To eliminate the spurious response above the anti-resonance frequency, we removed the reflector gratings portion in the conventional single-mode COM model. The admittance *Y* for the modified COM model changed from Equation (6) to $Y=P_{33}$. $P_{33}$ can be obtained from Equation (4). Then, we cascaded the traditional COM model with the modified COM model to obtain the ECOM model.

The requisite parameters for the ECOM model encompass the structural parameters and material parameters of the conventional single-mode COM model delineated previously, along with the material parameters corresponding to each of the spurious responses under consideration. The parameters used in this model were determined through numerical fitting.

### 2.4. Model Verification

**Figure 9:**
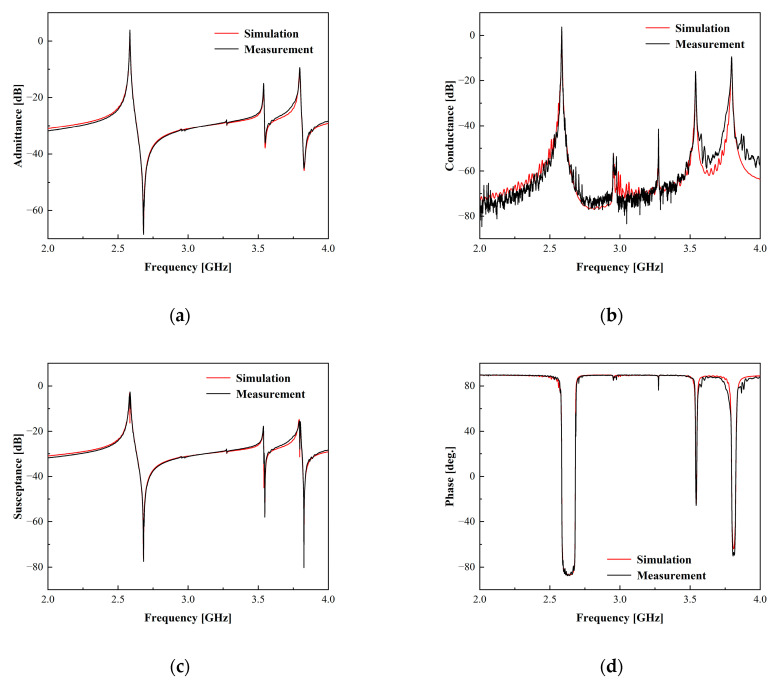
Comparative results of simulations using the ECOM model and measurements of one-port synchronous SAW resonator. (**a**) Simulated and measured admittance characteristics of a resonator. (**b**) Simulated and measured conductance characteristics of a resonator. (**c**) Simulated and measured susceptance characteristics of a resonator. (**d**) Simulated and measured admittance phase characteristics of a resonator.

To corroborate the efficacy of this model, we produced a one-port synchronous resonator using a 42°YX-LT thin piezoelectric layer. This resonator possessed the stacking structure illustrated in Figure 1 and adhered to the geometrical parameters delineated in Table 1. The ECOM model described in this study was utilized to simulate this resonator. Results of the simulation are presented in Figure 9 and compared with the measurement results from several perspectives of the one-port synchronous resonator in terms of the admittance, conductance, susceptance and phase. It is evident that the model simulation generally concurs with the measurements. The disagreement before the resonant frequency of each spurious response is due to the coupling between the spurious modes. Nonetheless, it is crucial to recognize that the aforementioned ECOM model successfully forecasted the high-frequency spurious responses.

**Figure 10:**
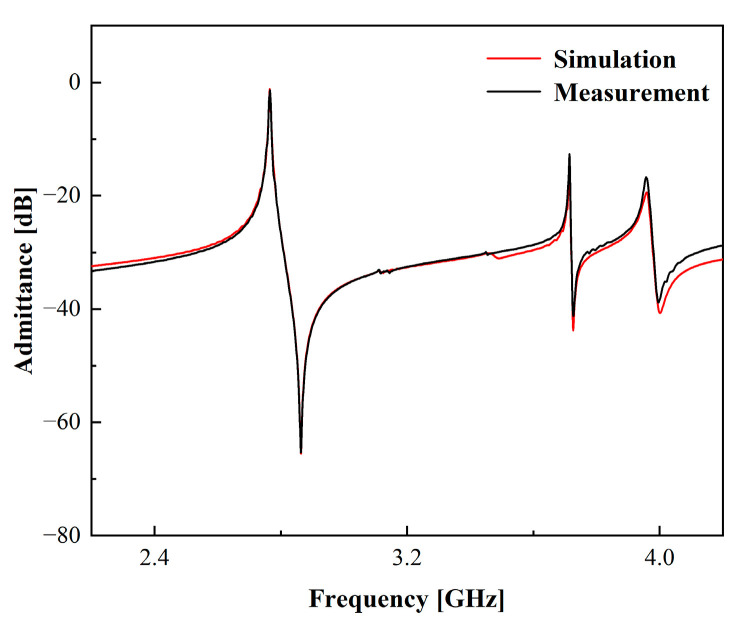
Comparative results of simulations using the ECOM model and measurements of one-port synchronous SAW resonator with 50°YX-LT as piezoelectric layer.

The model validation was primarily centered on the piezoelectric material 42°YX-LT. It is important to note that the ECOM model is also effective for other tangential piezoelectric materials, as demonstrated in Figure 10, which displays the validation experiments for the piezoelectric material 50°YX-LT. Table 2 delineates the structural parameters of the one-port synchronous resonator employed in this experimental validation. The ECOM model performs equally well for the piezoelectric substrate of 50°YX-LT. The impedance ratio of the high-frequency resonance at 3.45 GHz is a mere 0.5 dB, culminating in an error within the simulation. Nonetheless, such an error remains within acceptable bounds for the filter design process.

## 3. Results and Discussion

### 3.1. Simulation of Changing the Structural Parameters of the Resonators

Based on the rapid and precise simulation of one-port synchronous resonators using the ECOM model, this work confirms the ECOM model’s accuracy in modifying structural parameters in filter design. Furthermore, a ladder-type SAW filter was devised employing the ECOM model.

The ECOM model necessitates adjustments to be made to its structural parameters during the design of the ladder-type SAW filter so as to comply with the filter’s design requirements. Consequently, it becomes critical to verify the precision of the ECOM model subsequent to modifications in the structural parameters. In this paper, the structural parameters outlined in Table 1 serve as the parameters for the standard resonator. We aim to confirm the consistency between the ECOM model simulation and measurement by varying the aperture and the number of IDT finger pairs of the standard resonator. Initially, the ECOM model fitted the standard resonator, enabling us to obtain the structural and material parameters required for the model. Subsequently, we altered one of the structural parameters while maintaining the remaining structural and material parameters unaltered, thus acquiring the simulation results of the ECOM model post-parameter alteration. Eventually, we compared the simulation results with the measurement results to effectively confirm the precision of the ECOM model.

**Figure 11:**
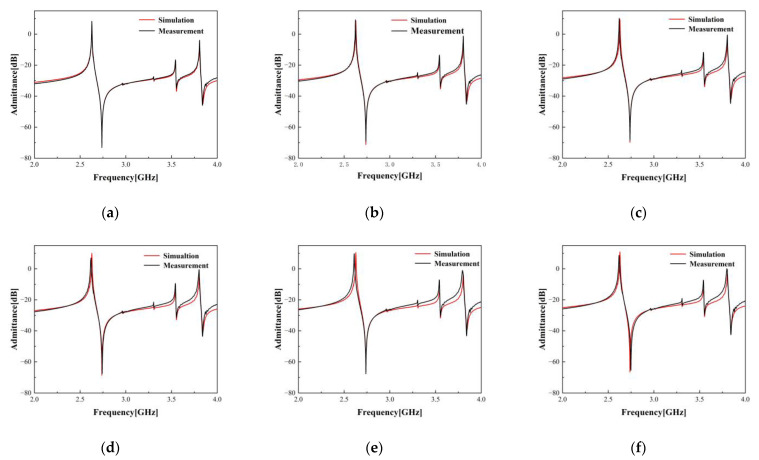
Simulation and measurement results of one-port synchronous resonator, with only the aperture altered while the other structural parameters remain constant. (**a**) The aperture W is 25 × *p*, and this resonator is used as the standard resonator. (**b**) The aperture W changed from 25 × *p* to 30 × *p*. (**c**) The aperture W changed from 25 × *p* to 35 × *p*. (**d**) The aperture W changed from 25 × *p* to 40 × *p*. (**e**) The aperture W changed from 25 × *p* to 45 × *p*. (**f**) The aperture W changed from 25 × *p* to 50 × *p*.

**Figure 12:**
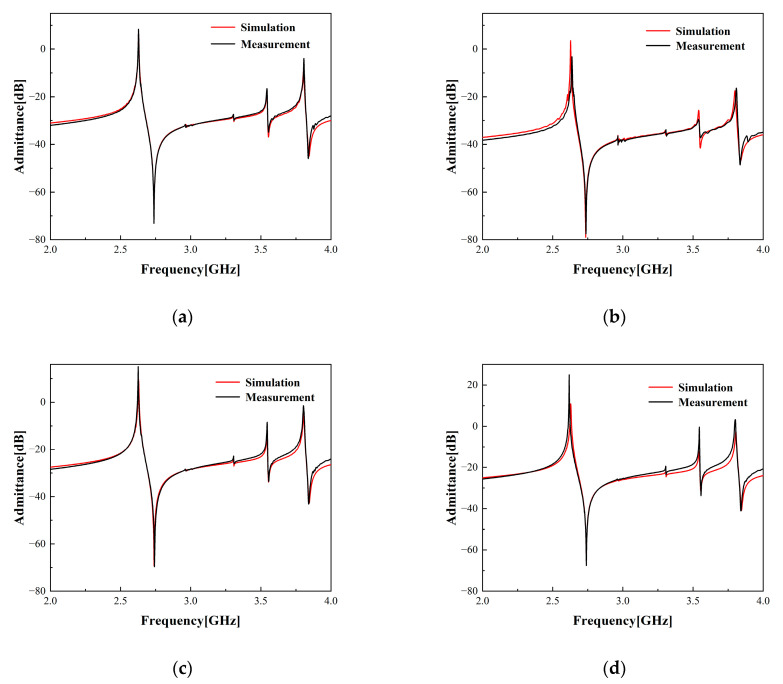
Simulation and measurement results of one-port synchronous resonator, with only the number of IDT finger pairs altered while the other structural parameters remain constant. (**a**) The number of IDT finger pairs $N_{t}$ is 100, and this resonator is used as the standard resonator. (**b**) The number of IDT finger pairs $N_{t}$ changed from 100 to 50. (**c**) The number of IDT finger pairs $N_{t}$ changed from 100 to 150. (**d**) The number of IDT finger pairs $N_{t}$ changed from 100 to 200.

Figure 11 illustrates a comparative analysis between the simulation and empirical measurement outcomes of the ECOM model upon modifying the aperture of the standard resonator. Table 3 depicts the structural parameters of the resonators used in this set of experiments. Figure 12 presents a comparison between the simulated and measured outcomes of the ECOM model subsequent to the alteration of the number of IDT finger pairs in the standard resonator. Table 4 lists the structural parameters of the resonators used in this set of experiments. As can be seen, the ECOM model’s simulation and measurement results align better when the aperture and the number of IDT finger pairs are varied between specific ranges. However, increasing the structural parameter variation widens the gap between simulation and measurement results. This is due to the inclusion of the resonator and wire effects in the actual measurements. When the variation in the structural parameters is larger, a larger variation in the wire area is required, and thus, there is a larger impact on the actual measurement results. Due to the requirements within the filter’s layout area and package, the structural parameters must be adjusted within a specific range. Therefore, the ECOM model’s accuracy is dependable during the filter design process.

### 3.2. Simulation of Ladder-Type SAW Filters

Following the comparative analysis with the basic resonator, an assessment of the actual filter device was undertaken. In this section, a comparison of the simulated and measured results using the ladder-type SAW filter is described.

The devices used in this paper were filter configurations employing a SAW resonator as a circuit element. This type of filter, called a ladder-type SAW filter or impedance element filter, offers low insertion loss and high-power durability, compared with acoustically coupled resonators [33,34]. For greater design flexibility, the resonators were used as an impedance element, while the filtering function was achieved through series and parallel resonator connections.

**Figure 13:**
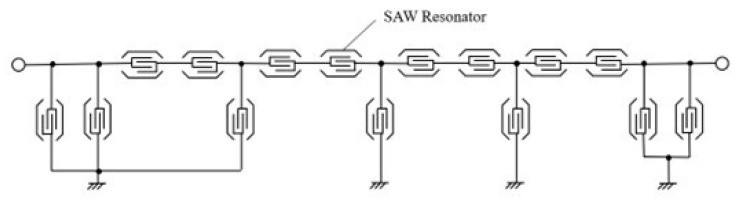
The topology of the ladder-type SAW filter used for measurements.

**Figure 14:**
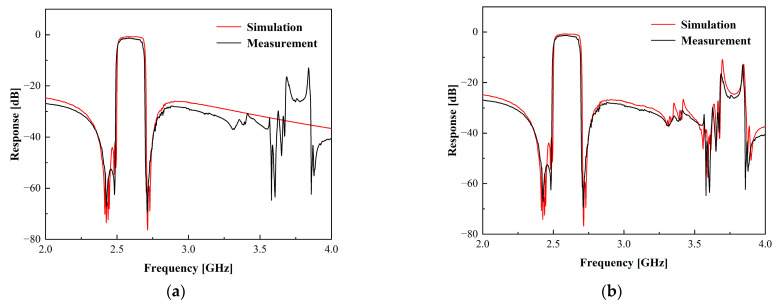
Measured and simulated filter response with (**a**) the traditional single-mode COM model and (**b**) the ECOM model.

Figure 13 shows the structure of the ladder-type SAW filter that was used for the experimental validation. To ascertain the disparity between the COM model and the ECOM model throughout the design process, an independent validation of the filter design outcomes was executed. Figure 14a shows the single-mode COM model. The measured result shows many spikes outside the high-frequency band of the filter; the simulated result could not predict them. Figure 14b shows the ECOM model while considering the high-frequency spurious responses. The result shows good agreement with the measurement. As the ECOM model extends the COM model, it incorporates its advantages. In this experiment, the structural parameters of both models were set to be the same to ensure accurate results comparison. Therefore, the simulation results of the ECOM and COM models were consistent below 3 GHz. In some high-frequency ranges, there will be a certain gap between the simulation and the measurement results due to the impact of the wire, the preparation process, etc. This gap is acceptable within the filter design process. From this study, it is clear that improvements to the model are important for the accuracy of predicting spurious responses. In addition, the ECOM model still has some limitations in the filter design process. Because the ECOM model is able to accurately describe multiple high-frequency spurious responses, the ECOM model requires more parameters than the traditional single-mode COM model, and therefore, the fitting process is more complicated. From the design efficiency point of view, the ECOM model is less efficient than the traditional single-mode COM model.

## 4. Conclusions

In this paper, we propose the ECOM model to precisely simulate and analyze the high-frequency spurious response of I.H.P. SAW devices. This facilitates the design process of SAW devices and avoids design errors resulting from imprecise high-frequency spurious response simulations.

First, the basic characteristics of an I.H.P. SAW device were analyzed by the traditional COM model and the P-matrix model. A comparison was made of the simulation and measurement results of the COM model for a one-port synchronous resonator. As a result, we realized the shortcomings of the COM model for simulating the high-frequency spurious response of I.H.P. SAW devices.

Then, the phenomenological model was modified, and the COM model underwent a simple extension. The ECOM model was proposed, which can successfully simulate the high-frequency spurious responses.

To verify the ECOM model, the one-port synchronous resonator and ladder-type SAW filter measurement were compared. The comparative analysis demonstrates that the ECOM model can accurately predict the high-frequency spurious responses. Furthermore, we varied the structural parameters of the ECOM model, such as the aperture diameter and the number of IDT finger pairs, and compared them with the real measurements. The findings suggest that the ECOM model fulfills the precision requirements within the filter design process. Therefore, the ECOM model is applicable in practical filter design tasks.

The ECOM model can predict the high-frequency spurious response of I.H.P. SAW devices relatively accurately, providing a reliable reference for designers during the design process.

## Figures and Tables

**Table 1:** The structural parameters for the one-port synchronous SAW resonators.

Parameters	Value
Period of the fingers *p*, (SAW wavelength λ)	1.4 (μm)
Metallic ratios α	0.5
Aperture W	25 × *p*
Number of IDT finger pairs $N_{t}$	100
Number of reflector gratings $N_{g}$	30

**Table 2:** The structural parameters for the one-port synchronous SAW resonators with 50°YX-LT as piezoelectric layer.

Parameters	Value
Period of the fingers *p* (SAW wavelength λ)	1.32 (μm)
Metallic ratios α	0.5
Aperture W	20 × *p*
Number of IDT finger pairs $N_{t}$	100
Number of reflector gratings $N_{g}$	30

**Table 3:** The geometric structure parameters of the resonators used to verify the accuracy of the ECOM model as the aperture changes, where the resonator with an aperture of 25 × *p* is designated as the standard resonator.

Parameters	Value
Period of the fingers *p* (SAW wavelength λ)	1.4 (μm)
Metallic ratios α	0.5
Aperture W	25 × *p* (standard resonator)
	30 × *p*
	35 × *p*
	40 × *p*
	45 × *p*
	50 × *p*
$\mathrm{Number}\;\mathrm{of}\;\mathrm{IDT}\;\mathrm{finger}\;\mathrm{pairs}\;N_{t}$	100
$\mathrm{Number}\;\mathrm{of}\;\mathrm{reflector}\;\mathrm{gratings}\;N_{g}$	30

**Table 4:** The geometric structure parameters of the resonators used to verify the accuracy of the ECOM model as the number of IDT finger pairs changes, where the resonator with the number of IDT finger pairs of 100 is designated as the standard resonator.

Parameters	Value
Period of the fingers *p* (SAW wavelength λ)	1.4 (μm)
Metallic ratios α	0.5
Aperture W	25 × *p*
Number of IDT finger pairs $N_{t}$	50
	100 (standard resonator)
	150
	200
Number of reflector gratings $N_{g}$	30

## Data Availability

The data presented in this study are available on request from the corresponding author. The data are not publicly available due to a non-disclosure agreement.

## References

[1] Weigel R., Morgan D.P., Owens J.M., Ballato A., Lakin K.M., Hashimoto K., Ruppel C.C.W. (2002). Microwave acoustic materials, devices, and applications. IEEE Trans. Microw. Theory Tech..

[2] Yang Y., Dejous C., Hallil H. (2023). Trends and Applications of Surface and Bulk Acoustic Wave Devices: A Review. Micromachines.

[3] Balteanu F., Modi H., Choi Y., Lee J., Drogi S., Khesbak S. 5G RF Front End Module Architectures for Mobile Applications. Proceedings of the 49th European Microwave Conference (EuMC).

[4] Sahu A., Aaen P.H., Devabhaktuni V.K. (2019). Advanced technologies for next-generation RF front-end modules. Int. J. Rf Microw. Comput.—Aided Eng..

[5] Chen P., Li G.X., Zhu Z.Y. (2022). Development and Application of SAW Filter. Micromachines.

[6] Hashimoto K.Y., Bao J.F. COM-Based Perturbation Analysis of Nonlinear Signal Generation in IHP SAW Resonators. Proceedings of the IEEE International Ultrasonics Symposium (IUS).

[7] Kimura T., Omura M., Kishimoto Y., Hashimoto K.Y. Applicability Investigation of SAW Devices in the 3 to 5 GHz range. Proceedings of the 2018 IEEE/MTT-S International Microwave Symposium-IMS.

[8] Nakagawa R., Iwamoto H., Takai T. Low Velocity IHP SAW Using Al/Pt Electrodes for Miniaturization. Proceedings of the IEEE International Ultrasonics Symposium (IUS).

[9] Takai T., Iwamoto H., Takamine Y., Nakao T., Hiramoto M., Koshino M.I.H.P. SAW technology and its application to micro acoustic components. Proceedings of the 2017 IEEE International Ultrasonics Symposium (IUS).

[10] Takamine Y., Takai T., Iwamoto H., Nakao T., Koshino M. A Novel 3.5 GHz Low-Loss Bandpass Filter Using IHP SAW Resonators. Proceedings of the Asia-Pacific Microwave Conference (APMC).

[11] Wong Y.P., Matsuoka N., Qiu L.Y., Hashimoto K. Analysis of SAW Slowness Shape on IHP SAW Structures. Proceedings of the IEEE International Ultrasonics Symposium (IEEE IUS).

[12] Wu X.Q., He Y.W., Shi B., Li W.P., Bao J.F., Hashimoto K.Y. A Simple Technique to Evaluate Lateral Leakage and Transverse Mode Behaviors of Reflectors in SH-Type SAW Resonator. Proceedings of the IEEE International Ultrasonics Symposium (IUS).

[13] Solal M., Gratier J., Aigner R., Gamble K., Abbott B., Kook T., Chen A., Steiner K. Transverse modes suppression and loss reduction for buried electrodes SAW devices. Proceedings of the 2010 IEEE International Ultrasonics Symposium.

[14] Iwamoto H., Takai T., Takamine Y., Nakao T., Fuyutsume T., Koshino M. Transverse Modes in IHP SAW Resonator and Their Suppression Method. Proceedings of the IEEE International Ultrasonics Symposium (IUS).

[15] Wong Y.P., He Y.W., Matsuoka N., Liang Q., Bao J.F., Hashimoto K. IHP SAW Transverse Edge Design for Energy Confinement with Suppressed Scattering Loss and Transverse Mode. Proceedings of the IEEE International Ultrasonics Symposium (IEEE IUS).

[16] Goto R., Fujiwara J., Nakamura H., Hashimoto K. (2018). Multimode coupling of modes model for spurious responses on SiO_2_LiNbO_3_ substrate. Jpn. J. Appl. Phys..

[17] Huang Y.L., Bao J.F., Li X.Y., Zhang B.F., Tang G.B., Omori T., Hashimoto K.Y. (2018). Influence of Coupling Between Rayleigh and SH SAWs on Rotated *Y*-Cut LiNbO_3_ to Their Propagations. IEEE Trans. Ultrason. Ferroelectr. Freq. Control.

[18] Mayer M., Ammann S., Pernpeintner M., Johnson J., Ebner T., Wagner K. Multi-mode P-matrix models for the description of interacting modes in TCSAW and LSAW devices. Proceedings of the IEEE International Ultrasonics Symposium (IUS).

[19] Tang G.B., Goto R., Nakamura H. Modeling and Suppression Method for Guided Mode in TC-SAW Devices. Proceedings of the IEEE International Ultrasonics Symposium (IUS).

[20] Pang J.Y., Wang S.B., Tang Z.F., Qin Y.M., Tao X.F., You X.H., Zhu J.K. (2022). A new 5G radio evolution towards 5G-Advanced. Sci. China Inf. Sci..

[21] Wang Z.Q., Du Y., Wei K.J., Han K.F., Xu X.Y., Wei G.M., Tong W., Zhu P.Y., Ma J.L., Wang J. (2022). Vision, application scenarios, and key technology trends for 6G mobile communications. Sci. China-Inf. Sci..

[22] Goyal A., Kumar K. LTE-Advanced Carrier Aggregation for Enhancement of Bandwidth. Proceedings of the International Conference on VLSI, Communications, and Signal Processing (VCAS), Motilal Nehru Natl Inst Technol Allahabad.

[23] Xu H.P., Fu S.L., Su R.X., Liu P.S., Wang R., Zeng F., Song C., Wang W.B., Pan F. (2023). Dual-Passband SAW Filter Based on a 32°YX-LN/SiO_2_/SiC Multilayered Substrate. Micromachines.

[24] Plessky V., Koskela J. (2000). Coupling-of-modes analysis of saw devices. Int. J. High Speed Electron..

[25] Ruppel C.C.W., Ruile W., Scholl G., Wagner K.C., Manner O. Review of models for low-loss filter design and applications. Proceedings of the 1994 Proceedings of IEEE Ultrasonics Symposium.

[26] Wu T.T., Wang S.M., Chen Y.Y., Wu T.Y., Chang P.Z., Huang L.S., Wang C.L., Wu C.W., Lee C.K. (2002). Inverse determination of coupling of modes parameters of surface acoustic wave resonators. Jpn. J. Appl. Phys. Part 1 Regul. Pap. Brief Commun. Rev. Pap..

[27] Wu Z.H., Liu Y.M., Shi B., Bao J.F., Hashimoto K.Y. COM-based Modeling of SAW Scattering at Reflector Outer Edges in IHP SAW Resonator. Proceedings of the IEEE International Ultrasonics Symposium (IUS).

[28] Morgan D.P. (1996). Cascading formulas for identical transducer P-matrices. IEEE Trans. Ultrason. Ferroelectr. Freq. Control.

[29] Wagner K., Mayer M., Bergmann A., Riha G. A 2D P-Matrix Model for the Simulation of Waveguiding and Diffraction in SAW Components. Proceedings of the IEEE Ultrasonics Symposium.

[30] Ash E.A. Surface Wave Grating Reflectors and Resonators. Proceedings of the G-MTT 1970 International Microwave Symposium.

[31] Hashimoto K., Endoh G., Yamaguchi M. Coupling-of-modes modelling for fast and precise simulation of leaky surface acoustic wave devices. Proceedings of the 1995 IEEE Ultrasonics Symposium—Proceedings—An International Symposium.

[32] Naumenko N., Abbott B. Hybrid surface-bulk mode in periodic gratings. Proceedings of the IEEE International Ultrasonic Symposium.

[33] Komatsu T., Tanaka Y., Hashimoto K.Y., Omori T., Yamaguchi M. (2009). Design of narrow bandwidth ladder-type filters with sharp transition bands using mutually connected resonator elements. IEEE Trans. Ultrason. Ferroelectr. Freq. Control.

[34] Ruppel C.C.W. (2017). Acoustic Wave Filter Technology-A Review. IEEE Trans. Ultrason. Ferroelectr. Freq. Control.

